# BPM Support for Patient-Centred Clinical Pathways in Chronic Diseases

**DOI:** 10.3390/s21217383

**Published:** 2021-11-06

**Authors:** Marek Szelągowski, Justyna Berniak-Woźny, Cezary Lipiński

**Affiliations:** 1 Systems Research Institute, Polish Academy of Sciences, Newelska 6, 01-447 Warsaw, Poland; marek.szelagowski@dbpm.pl; 2 Center for Innovation and Technology Transfer, Medical University of Lodz, 90-149 Łódź, Poland; cezary.lipinski@umed.lodz.pl

**Keywords:** business process management (BPM), telemedicine, Chronic Obstructive Pulmonary Disease (COPD), clinical pathways (CPs), diagnostic and therapeutic processes

## Abstract

Epidemiological trends over the past decade show a significant worldwide increase in the burden of chronic diseases. At the same time, the human resources of health care are becoming increasingly scarce and expensive. One of the management concepts that can help in solving this problem is business process management (BPM). The results of research conducted in the healthcare sector thus far prove that BPM is an effective tool for optimizing clinical processes, as it allows for the ongoing automatic tracking of key health parameters of an individual patient without the need to involve medical personnel. The aim of this article is to present and evaluate the redesign of diagnostic and therapeutic processes enabling the patient-centric organization of therapy thanks to the use of new telemedicine techniques and elements of hyperautomation. By using an illustrative case study of one of the most common chronic diseases, Chronic Obstructive Pulmonary Disease (COPD), we discuss the use of clinical pathways (CPs) prepared on the basis of the current version of the Global Initiative for Chronic Obstructive Lung Disease (GOLD) as a communication tool between healthcare professionals, the patient and his or her caregivers, as well as the method of identifying and verifying new knowledge generated on an ongoing basis in diagnostic and therapeutic processes. We also show how conducting comprehensive, patient-focused primary health care relieves the health care system, and at the same time, thanks to the use of patient engagement and elements of artificial intelligence (predictive analyses), reduces the significant clinical risk of therapy.

## 1. Introduction

The challenge facing the healthcare sector is to provide services of better quality that are more tailored to the individual needs of patients. This requires a redesign of diagnostic and therapeutic processes to enable a holistic view of the patient’s health and disease, and simultaneously enable the most effective use of available resources. To achieve the best cost/quality ratio, healthcare management must follow the same path as other sectors and therefore apply similar concepts, methods, and technologies. One of the most effective and most frequently used concepts today is business process management [1]. The results of research conducted in the healthcare sector thus far prove that BPM is a feasible and effective tool for optimizing clinical processes, as it allows for the ongoing automatic tracking of key health parameters of an individual patient without the need to involve medical personnel [2,3,4]. To expand and accelerate change and harness the potential of BPM in the health care sector, greater acceptance and commitment from the clinical community is needed, as well as stronger experimental research to test its actual and optimal scope of application [3,5]. It is also necessary to be accepted by patients and their caregivers, who are not the object but the subject of diagnostic and therapeutic processes. Without their cooperation, even the best and most innovative methods of diagnostics and therapy will not translate into better results.

Chronic diseases already affect one third of the European population aged 15 and over [6] and 23.5% of Europeans of working age [7]. Their treatment is long-term and expensive and its effects usually depend on the coordination of therapeutic activities of interdisciplinary teams (often varying from a few to several dozen members) and the attitudes of the patients themselves. The contemporary view on disease and health management also draws increasing attention to the need to engage the patients and their caregivers in the therapeutic process. This trend is increasingly supported by changes in the social culture and the widespread availability of telemedicine technology that enables remote, real-time tracking and the prediction of changes in an increasing number of parameters critical to patient health. However, its use requires a change in traditional roles in the therapeutic process, according to which the doctor treats and the patient undergoes therapy. Especially in the case of chronic diseases, conscious and voluntary (without force or coercion) involvement of the patients and their caregivers in the treatment of the disease is essential. This requires specialists to provide the patient with knowledge about the therapy in a clear and understandable form and to clearly define the scope of the patient’s responsibility. It also requires continuous updating of medical knowledge and continuous analysis of the patient’s critical vital signs and updating their individual care plan (ICP) as needed. The care pathway approach offers interdisciplinary care teams the same focus, with roles and communication channels set and adhering to the same evidence-based standards.

The aim of this article is to present and evaluate the redesign of diagnostic and therapeutic processes enabling patient-centric organization of therapy with the use of new telemedicine techniques and elements of hyperautomation. This is particularly important today for two reasons. First, the COVID-19 pandemic has shown that skilled and committed workers are the most critical healthcare sector resource, and how quickly these resources are depleted when poorly managed. Secondly, it caused significant changes in the social and work culture, as well as in health care, as a result of which the acceptance of the implemented telemedicine technologies and elements of hyperautomation abruptly increased. However, taking advantage of the emerging opportunity to increase quality, reduce costs, and improve the cost/quality ratio requires a redesign of health care processes and a change in the approach to protecting the health of patients and their caregivers. In this article, for the presentation of models of clinical pathways, as an example of a chronic disease, we used Chronic Obstructive Pulmonary Disease (COPD)—currently one of the three leading causes of death in the world, which is responsible for over 3 million deaths annually [8]. COPD is also the most common chronic respiratory disease in adults [8]. It is estimated that around the world, 11.7% of people over 40 suffer from COPD [8]. Due to aging populations and increasing environmental threats, the number of patients suffering from chronic diseases is expected to increase. Although COPD is a preventable and treatable disease, its course is often fraught with exacerbations, the presence of which usually greatly accelerates the progression of the disease itself. Unfortunately, it is estimated that the disease is diagnosed at an early stage in less than 20% of patients. Hence, a significant population of patients is not diagnosed or is diagnosed only in the advanced stage of the disease [9]. Moreover, COPD is an additional, very serious risk factor when a patient suffers from COVID-19 [8].

The design of the clinical pathway model requires a detailed understanding of the COPD diagnosis and treatment process. For this reason, the clinical pathway was developed on the basis of the Global Initiative for Chronic Obstructive Lung Disease (GOLD) report, updated in 2021 [8], containing the latest medical knowledge related to the COVID-19 pandemic and taking into account the current possibilities of using telemedicine technology. Problems and inefficiencies in the clinical pathway were identified and addressed in the iterative design process and consulted with two high-class experts in the field of COPD from the Lodz Medical University, as we wanted to get a broader view of the complex clinical process and cover patients’ and their caregivers’ full engagement, as well as the newest and quickly changing telemedicine technology, integrating events and changes for each individual situation. Such an approach would improve the quality of work performed by healthcare professionals and reduce their workload.

### 1.1. Changes in the Social and Work Culture of Health Care Personnel

Telemedicine is defined as medical care provided remotely to a patient in a separate location using two-way voice and/or visual communication (as by computer or smartphone) [10]. The barrier in direct contacts created by epidemiological recommendations during the COVID-19 pandemic resulted in a leap in the acceptance of telemedicine among medical personnel as well as among patients and their caregivers [11]. Difficulties in direct contacts caused rapid changes in the social and work culture of health care workers, thanks to which the advantages of telemedicine were appreciated, such as [12,13]:

- Remote contact, no need to spend time and costs in reaching the visit/consultation by the patient or medical staff;
- Possibility of remote consultations with specialists unavailable in the patient’s place of residence;
- The ability to monitor the patient’s health online without the need to hospitalize the patient;
- The possibility of collecting and analysing data sets characterizing the patient’s condition in selected periods of time without the need for hospitalization;
- The possibility of using predictive analytics and artificial intelligence to prevent foreseeable acute states;
- Improvement of the quality of services provided thanks to the use of elements of telemedicine and hyperautomation.

The health care system noticed the benefits of using telemedicine in the form of reducing the workload, number and length of hospitalizations, shortening the time of reaching and contact with the patient, allowing for better time management of health care workers and reducing costs. In the COVID-19 Healthcare Coalition Telehealth Workgroup study, more than three-quarters of physicians surveyed said telemedicine helped them provide better care for patients and more than 80% said that their patients have reacted favourably to using telehealth for care [14]. This seems to be a good prognosis for the development and successful implementation of telemedicine services, and even the extension of their scope and enrichment of communication channels with video layers and signal transmission from ECG-type measuring devices, pulse oximetry, auscultation of the lungs and bronchial tree, etc. [15,16,17].

### 1.2. Clinical Pathways as Patterns of Knowledge-Intensive Business Processes

The use of BPM requires a holistic view and orientation of the implemented activities towards their goals, a clear definition of responsibility and empowerment of all participants, as well as the continuous accumulation of knowledge and process improvement [18]. For the purposes of health care, traditional process management is insufficient, as it assumes the possibility of accurately designing the course of a process based on the full knowledge of its course before its implementation. The health care system undoubtedly requires periodic reassessment and flexibility due to constant changes in clinical guidelines and the variability of the patient’s condition, which leads to the model being permanently adapted and improved upon. Due to the unpredictability and dependence of the results of diagnostic and therapeutic processes on the use of knowledge in a specific context of process implementation, it is necessary to use dynamic business process management (dynamic BPM) to describe and implement diagnostic and therapeutic processes in the most effective way [5,19].

Diagnostic and therapeutic processes, also known as clinical pathways (CPs) [20,21] or care chains [22], are care maps designed to improve the quality of personalized patient care and standardize care procedures while establishing cost-effective evidence-based care management. They are usually described at three levels of detail:

Level I—National and international guidelines developed by teams of specialists on the basis of their own knowledge and knowledge gathered in national and international knowledge bases;

Level II—Clinical pathways created at the level of the health care unit on the basis of relevant national and international guidelines as well as information about the resources, skills and competences of its staff;

Level III—Individual Plan of Care (IPC) developed on the basis of level II clinical pathways, for a specific patient and taking into account the specific context of therapy, including patient age, health condition, comorbidities, possibilities and limitations of therapy.

Kinsman, Rotter, James, Snow and Willis [23] conducted a literature review and developed detailed criteria for what should be classified as a clinical pathway (CP) and verified this against 260 papers. They developed the following criteria:

- The intervention was a structured multidisciplinary plan of care;
- The intervention was used to channel the translation of guidelines or evidence into a local organization and its structures;
- The intervention detailed the steps in a course of treatment or care in a plan, pathway, algorithm, guideline, protocol, or another inventory of actions;
- The intervention had time-frames or criteria-based progression (that is, steps were taken if designated criteria were met).

The intervention aimed to standardise care for a specific clinical problem or episode of healthcare in a specific population. If an intervention satisfied the first, and then any two of the remaining three criteria, then it was classified as a clinical pathway.

When implementing BPM in healthcare, it should be kept in mind that:

- The patient is treated, not the disease;
- The patients and their caregivers should be involved in the treatment process;
- Different clinical and peri-clinical pathways (management, support, logistics and others) must form a coherent system;
- Knowledge available in the form of clinical pathways should be available and be reflected in IT and telemedicine technology used on a daily basis by all stakeholders of the diagnostic and therapeutic process.

The implementation of CPs requires the empowerment of process executors to make diagnostic and therapeutic decisions in accordance with the possessed knowledge and the requirements of the clinical context of process implementation. These decisions have a significant impact on the course and value provided by the implemented processes [24]. Obviously, the greater the knowledge and commitment of both the patients and their caregivers and the therapeutic team, the lower the risks during the process and the greater the chance that it will achieve its goals.

**Figure 1:**
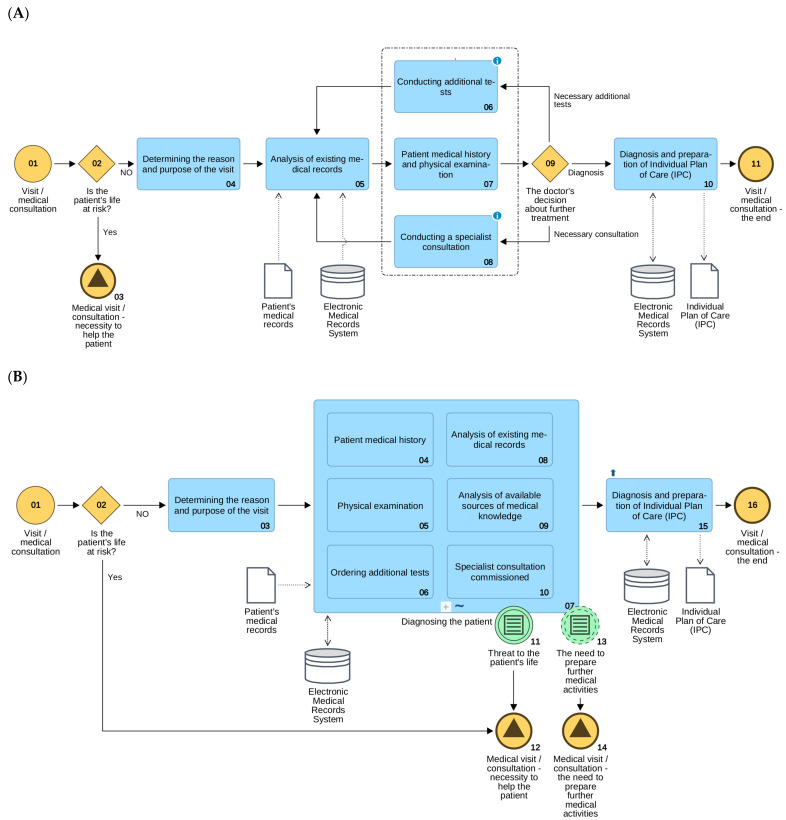
Diagram of the process visit/medical consultation: (**A**) static (structured) model; (**B**) dynamic model (by ADONIS).

Diagnostic and therapeutic processes are typical knowledge-intensive business processes (kiBPs), because their result is strictly dependent on the use of knowledge of all participants in the process [25]. Due to their unpredictability, it is necessary to take into account that at the time of modelling and designing, full, detailed knowledge on how to execute them is not available. Therefore, regardless of the form of the description of CPs, it is necessary to take into account the possibility of dynamic management of the process flow, as well as make decisions unforeseen by the CP designer. This makes it impossible to design clinical pathways like traditional business processes as a sequence of mandatory actions (Figure 1A). The CP model must enable executors (including the patient) to adjust the process flow to the specific context of execution, including events occurring, equipment resources available, available specialists and drugs, etc. [19].

According to the CP model shown in Figure 1B, under the sub-process “Diagnosing a patient,” the diagnostician may perform the presented six tasks in any order and each of them may be performed any number of times, depending on the patient’s diagnostic needs.

The process ends when the diagnostician decides that he or she has obtained enough information to make a diagnosis according to his or her knowledge. Each execution of the process may therefore have a different course (a different sequence of performed tasks) depending on the patient’s condition, the doctor’s knowledge, the medical documentation at the beginning, available diagnostic tests, the possibility of consultations or the occurrence of unforeseen events, such as the need to provide immediate assistance to the patient. Not only does the analysis of completed CPs allow for the verification of the correctness of the therapy, it also allows for the verification of the usefulness and effectiveness of the knowledge used, as well as the creation of new knowledge based on the analysis of individual cases and the identification and analysis of deviations from the standard clinical path [26,27]. Such an analysis of hundreds or thousands of CP implementations is in practice impossible without the use of process mining techniques to discover the course of processes or artificial intelligence (AI) for a detailed analysis of their course and results. Looking from the BPM perspective, CPs and BigData collections of medical records of the implemented diagnostic and therapeutic processes play the role of a repository of knowledge, simultaneously serving to verify the old, but also to identify new knowledge obtained directly from the data of conducted therapies [18].

### 1.3. Chronic Disease Management

The Centers for Disease Control and Prevention (CDC) define chronic diseases as conditions that last 1 year or more and that require ongoing medical attention or limit activities of daily living, or both [28]. These are progressive diseases which, according to current medical knowledge, cannot be cured. During the long-term course of these diseases, they significantly, usually progressively, impair the functioning of those affected and their environment. The disease, which lasts for years (and sometimes decades), presents the patients and their caregivers with increasing challenges in everyday life, but also enables them to significantly influence the effectiveness of ongoing therapy. As the disease progresses, the influence of the daily involvement of the patient and their caregivers on the effectiveness of therapy is becoming increasingly apparent. It can be implemented through an appropriate lifestyle, diet, exercise and rehabilitation, consistent implementation of drug therapy, prevention of exacerbations, etc. [29]. Although it is not currently possible to cure chronic diseases, it is possible to slow down or stop their progress and improve the quality of life of patients and their environment. However, this requires actual, conscious cooperation of all participants in the therapeutic process [16,30]. For obvious reasons, it is impossible to achieve positive effects of therapy without the commitment and co-responsibility of the patients and their caregivers. This requires giving them the role of an active participant in the therapeutic process, which changes their current position from a passive recipient to an active participant or even a co-creator of the therapy. It also helps the treatment team collect information and modify the IPC based thereon [31,32,33]. This document should include:

- A level III CP developed on the basis of the applicable guidelines (CP level I and II) in a way that individually adjusts the therapy to a specific patient, taking into account the broad clinical background, including parallel diagnostic and therapeutic procedures in the field of comorbidities;
- Clear definition of goals and parameters used to assess the implementation of tasks set at specific stages of the therapeutic process;
- A clear and understandable description of the forms of medical care planned for implementation and the manner of patient participation in them, so that the patient is aware of the conditions and what activities and therapeutic activities should be undertaken, in what situations he or she should consult the attending physician, and what forms of activity should be strictly abandoned or avoided.

In line with the postulates of coordinated medicine and personalized medicine, IPC is therefore a kind of agreement between the therapeutic team and the patient in terms of mutual obligations of both parties, but at the same time a fundamental element of the health education of the patients and their caregivers [8,34]. The IPC of a chronic disease under the responsibility and validation of the patient should clearly indicate at what times what parameters the patient should measure, as well as how to present them to whom report them, and what levels of these parameters are recommended [32]. Thanks to the advances in telemedicine technologies, the patient can increasingly often perform the tasks specified in the IPC in an autonomous manner, without involving a doctor or other medical personnel. At the same time, based on generally available cloud computing technologies, it is possible to automatically collect and transfer data and, if required, ensure their anonymity.

## 2. Materials and Methods

The aim of this article is to show how BPM methods have been used to develop integrated and inclusive clinical pathways in the treatment of one of the most common chronic diseases—COPD. An illustrative case study presents an example of coherent CPs dedicated to COPD, which are implemented at the Clinical and Didactic Center of the Medical University of Lodz and depict how the clinical pathway should be organized to provide the patient with effective treatment and engagement was used for this purpose.

The illustrative case study is descriptive, in-depth and rich in context to provide the reader with visually descriptive details that are important to support the research process and the understanding of its outcomes [35]. When applying an illustrative case study, a small number of cases (one or two) should be used [36]. In this paper, a single case study is applied to gain insight into the organization of a complex care system of the pathway. It aims to serve as a guideline for interdisciplinary teams of health professionals, patients, and their caregivers and employ the newest telemedicine technology to maximize the treatment effectiveness and create the knowledge that upgrades the efficiency of the healthcare system. Due to its complexity and deviation from routine clinical pathways, it is an extremely interesting case to explore [37,38].

## 3. Results: Clinical Pathways for Chronic Obstructive Pulmonary Disease

A point of departure for the analysis of the course of this disease, as well as its diagnostics and treatment, rests in the current treatment recommendations of COPD, containing detailed diagnostic and therapeutic information, from the Global Initiative for Chronic Obstructive Lung Disease [8]. This document is being updated annually.

In GOLD, considerable stress is put on broadening the role of the patient with respect to controlling their symptoms and making independent preventative and therapeutic actions, without the need of directly consulting medical doctors each time. For this reason, an IPC should be prepared with the participation of the patient. Should any doubt arise, the IPC should be negotiated among the patient and the therapist. In order to ensure effective and systematic cooperation among the patients and their caregivers and the members of the therapeutic team, it is crucial to use a tool which is understood by both sides with respect to evaluating the patient’s actual state and comparing it to the intended state. CPs fulfil these conditions, as they describe in a clear and concise fashion the logic of the proposed therapy, the tasks of the particular participants and crucial parameters pertaining to the patient’s state of health, which require control and which may result in even sudden changes to the methods used in the course of therapy. When the patient is empowered to consciously and actively participate in the diagnostic-therapeutic process with the use of CPs or the correlated IPC, he or she gains insight into the activities expected of them and is able to analyse them and undertake them. In turn, members of the therapeutic team gain the possibility of analysing the activities undertaken in the patient’s environment at selected time intervals and, with the use of telemedicine devices, also in real time. By having knowledge on his or her disease and understanding the therapeutic activities consciously, the patient manages his or her disease and contacts the indicated members of the therapeutic team in situations which require such contact, while also being able to find answers to questions and doubts without the need for contact in situations foreseen and described in the CP and the IPC.

**Figure 2:**
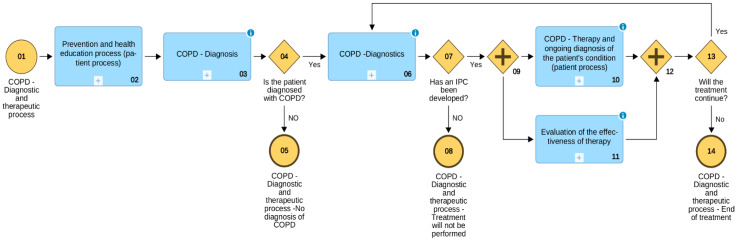
General map of the diagnostic and therapeutic process of COPD (by ADONIS).

As an example, the authors chose CPs COPD prepared at the Clinical and Didactic Center of the Medical University of Lodz. It is the main hospital in the agglomeration and province of Łódź, responsible for the treatment of patients with COVID-19 and patients with complications and consequences after undergoing COVID-19. Especially during the second and third wave of the pandemic, like most hospitals in Poland, the hospital suffered from a chronic shortage of specialist medical personnel and, due to the increased occurrence of pulmonary diseases resulting from COVID-19, especially personnel with knowledge on diagnosing and treating pulmonary diseases such as COPD or idiopathic pulmonary fibrosis (IPF). This was an additional impulse to develop a map of processes in accordance with the GOLD [8] recommendations, in which have clearly indicated ”patient processes,” whose initiator and the main person responsible for their course is the patient (Figure 2).

**Figure 3:**
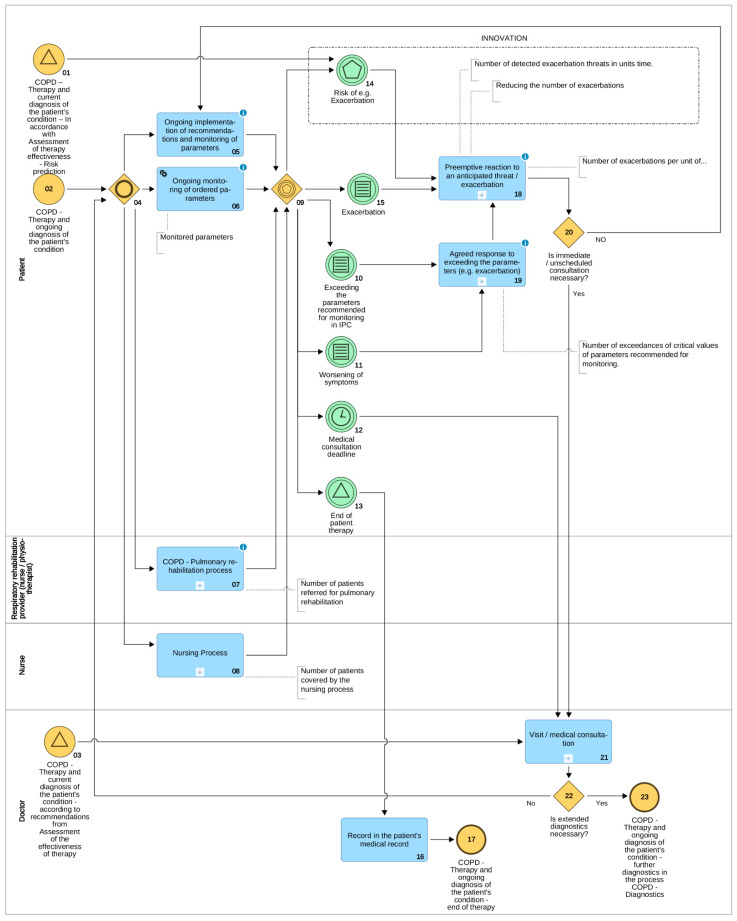
Process COPD—Therapy and ongoing diagnosis of the patient’s condition—patient process, in which the doctor and others (only) participate (by ADONIS).

Traditional COPD diagnostics processes (subprocesses no. 03 and 06 in Figure 3) are executed by the medical personnel. However, the Prevention and health education process, including preventative measures (no. 02), as well as COPD—Therapy and ongoing diagnosis of the patient’s condition process (no. 10) require not just the passive participation, but the active, conscious participation of the patients and their caregivers. As the COPD—Therapy and ongoing diagnosis of the patient’s condition process diagram shows (Figure 3), the focal point thereof is the patient’s performance of the task Ongoing implementation of recommendations and ongoing monitoring of parameters (tasks no. 05 and 06). The patient undergoing the treatment in accordance with the IPC is the one responsible. Of course, the patient can (and in present conditions should) be supported by telemedicine technology. Whenever possible, the use of telemedicine technology should accompany the automatic task Ongoing monitoring of ordered parameters. In this way, it is possible to control crucial IPC parameters of the state of the patient on an ongoing basis, such as blood pressure and saturation, or blood sugar level, which allows for the instant signalling of exceeding crucial parameters set in the IPC to caregivers or medical personnel.

In addition, it is possible, regardless of the patient’s condition, to signal health and life-threatening situations, such as sudden heart failure or fall of the monitored patient. Data collected in devices or online is sent to medical systems to enable therapists to track changes in important health parameters of the patient on an ongoing basis.

When using methods from the fields of machine learning (ML) or artificial intelligence (AI) in the treatment of COPD, ongoing analysis of the obtained data allows for the prediction of the state of the patient’s health, including upcoming exacerbations (event no. 14). It allows the patient and the doctor to undertake preventative steps to eliminate or mitigate threats (subprocess no. 18). As a result of taking preventive measures, consultation with a doctor is necessary only in some cases (gateway no. 20 and subprocess no. 21). Only as a result thereof, a decision about the necessity of hospitalization can be made (gateway no. 22 and final event no. 23). Thanks to the ongoing monitoring of the parameters of the patient’s condition, which does not burden medical personnel, it is possible to significantly reduce the number of hospitalizations, which relieves the health care system, and at the same time significantly minimizes the risks associated with hospitalization of a patient with COPD [13].

**Figure 4:**
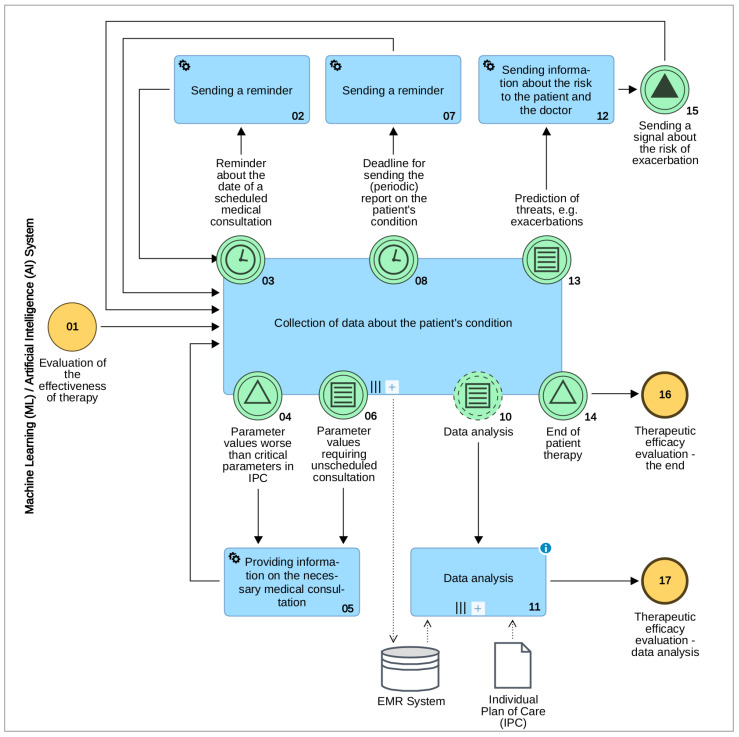
Diagram of the process Evaluation of the effectiveness of therapy (by ADONIS).

This possibility is particularly significant when the therapy is overseen by non-specialist doctors, who do not have deep knowledge of COPD, e.g., general practitioners. As shown on the diagram of the process Evaluation of the effectiveness of therapy performed in the system with elements of ML/AI (Figure 4) on the basis of the IPC (e.g., dates of planned visits) and ongoing data from telemedicine devices, the AI algorithm analyses data on an ongoing basis and communicates the recommended course of action to the patient without the participation of the medical doctor (subprocess no. 09 Collection of data about the patient’s condition). Concurrently, on-line analysis of the collected data allows for the automatic signalling of the need for a doctor appointment (task no. 05).

In the therapy of COPD as a chronic disease, the use of patient-centred CPs using telemedicine technologies allows for:

- Raising the feeling of security of patients and their caregivers;
- Lifting the burden off of the healthcare system by eliminating visits/medical consultations which are deemed non-essential according to the adopted IPC;
- On the basis of data collected and analysed on an ongoing basis, the prevention of exacerbations, which usually permanently deteriorate the state of the patient and which may also consist a risk of death;
- An ex post analysis on the basis of collected data of the introduced therapies with a view to verifying existing, as well as creating new fact-driven medical knowledge.

## 4. Discussion and Conclusions

In the healthcare system, the use of BPM methodologies allows for the ongoing automatic tracking of key parameters of the individual patient’s condition without the need for commitment to expensive medical resources. For the patients, it provides a chance for more effective treatment and better quality of life thanks to better adjustment of therapy and recommended pro-health behaviours to a specific clinical condition and, which is particularly important, through the conscious involvement of the patient and his or her caregivers in the effective reduction of disease risks and controlled self-management of the disease. This is of particular significance in the case of chronic diseases such as COPD, as it may lead to a reduction in possible exacerbation, slow disease progression and even stop the disease, thereby significantly reducing the social and healthcare costs. However, such a solution requires viewing the patient as an equal participant of the therapeutic process; as someone with rights, including the right to medical education and the right to co-decide on the course of therapy. The patients are required to depart from their traditional passive role in therapy and to acknowledge that they also have certain responsibilities and that their realization has an essential influence on the results of therapy. This also requires, particularly in light of the COVID-19 pandemic, to use telemedicine technology in order to lift the burden off of the healthcare system and raise the pace of creating knowledge helpful in combating the pandemic. The use of IT technologies already available in healthcare, such a cloud computing, BigData, process mining, predictive analytics, ML or AI allows for the real-time collection and analysis of data and their use with a view to verifying existing and creating new knowledge directly in the course of performing diagnostic-therapeutic processes. However, this requires making certain adaptations to existing healthcare rules and regulations to allow for performing and financing such actions in accordance with the proposed model.

Although the paper lacks broader quantitative studies that would allow for the assessment of the economic and therapeutic effects of, as well as the impact on the development of medical knowledge in the field of COPD treatment, it illustrates how standardised CPs can serve as guidelines for interdisciplinary teams of health professionals, patients and their caregivers and employ the newest telemedicine technology to maximize the treatment effectiveness and create knowledge that raises the efficiency of the healthcare system.

One crucial limitation of the work is its conceptual character. Despite the fact that telemedicine technologies are already available and the current version of GOLD refers to the treatment of COPD during the COVID-19 pandemic, further studies are necessary to explore the effectiveness of the proposed CP and its influence on raising the pace of development of medical knowledge. A particularly crucial area of future work, which will have considerable influence on the effectiveness of COPD treatment, is the data-driven analysis of the causes or signals of exacerbations. The authors and the pulmonologists involved in the study assume that on the basis of analyses of sets of BigData from completed treatments, new variants of CPs will be proposed with a view to mitigating the risk of exacerbations. At the same time, economic analysis pointing to the possibility of lowering direct costs of COPD treatment (e.g., hospital visits due to exacerbations) and long-term costs (e.g., the social costs of the patients’ exit from the labour market or the costs of long-term care) will result in rapid changes to healthcare rules and regulations and will allow for their broad use.
